# Breast elastography—ready for prime time?

**DOI:** 10.1007/s00330-023-10329-4

**Published:** 2023-10-26

**Authors:** André Pfob, Michael Golatta

**Affiliations:** 1 Department of Obstetrics and Gynecology, Heidelberg University Hospital, Im Neuenheimer Feld 440, 69120 Heidelberg, Germany; 2 National Center for Tumor Diseases (NCT), German Cancer Research Center (DKFZ), Heidelberg, Germany; 3 Brustzentrum Heidelberg, Klinikum Sankt Elisabeth, Heidelberg, Germany

The imaging community has evaluated the use of breast elastography for over 20 years. Elastography is an imaging technique that assesses the stiffness of a lesion and builds on the observation that, generally, carcinomas are stiffer than benign tissue [1]. However, breast elastography has not yet been widely accepted by the breast imaging community although the potential has been shown in several studies.

The non-adoption of breast elastography is largely associated with differences in methodology between vendors, variations in technique, and unclear clinical implications. Two general techniques in breast elastography can be distinguished: shear-wave elastography, a quantitative method to measure the lesion stiffness via the propagation of shear-waves, and strain elastography, a relative method that compares the lesion stiffness with the surrounding tissue [2]. Both shear-wave and strain elastography can be interpreted in different ways. For strain elastography, the elastography to B-mode length ratio (E/B ratio) has been established to be the most accurate interpretation method [3]. For shear-wave elastography, however, the appropriate interpretation technique with regard to the optimal cutoff is yet to be defined.

We observed a similar process with the introduction of 2D shear-wave elastography for liver stiffness evaluation in chronic liver diseases, where difficulties were finally overcome by a combination of standardizations between vendors and large prospective trials to drive clinical implications.

In this issue of *European Radiology*, Xu et al report the results of a large prospective, multicenter trial that evaluates the diagnostic performance of shear-wave elastography in combination with standard breast ultrasound for breast cancer diagnosis [4]. A total of 897 patients with BI-RADS 3 to 5 breast masses underwent both B-mode breast ultrasound and shear-wave elastography; histopathologic evaluation was conducted in those with BI-RADS 4 or 5 masses and a 2-year follow-up for those with BI-RADS 3 masses (upon standard B-mode breast ultrasound). The additional use of shear-wave elastography was evaluated by hypothetically reclassifying participants into the respective BI-RADS categories. The results confirm that a large proportion of patients, 46%, receives an (ultimately) unnecessary biopsy based on B-mode ultrasound as histopathology turns out to be benign. (Hypothetically) adding 2D + 3D shear-wave elastography reduces the proportion of benign biopsies compared to standard breast ultrasound by 54%. Specifically, BI-RADS 4a masses were downgraded to BI-RADS 3 with a cutoff of 90 kPa (= 5.5 m/s or less) and BI-RADS 3 masses were upgraded to BI-RADS 4a with a cutoff of 120 kPa (= 6.3 m/s or more); positive-predicative values (PPV) were 53.9% for B-mode breast ultrasound and 71.4% for the combination with shear-wave elastography. The proportion of missed malignancies was kept below the 2% threshold of the BI-RADS 3 category.

These results are in line with two other prospective multicenter trials. The multicenter BE1 study by Berg et al suggested cutoff values for downgrading BI-RADS 4a lesions at 80 kPa (= 5.2 m/s or less) and for upgrading BI-RADS 3 at 160 kPa (= 7.3 m/s or more); an improvement in specificity with the same sensitivity was observed (PPV 52.6% vs. 65.7%) [5]. A later prospective, multicenter, international study by Golatta et al could not confirm the exploratory cutoff values of previous studies, since the rate of false-positive findings was reduced, but at the expense of an increased rate of missed cancers. Secondary analyses suggest to downgrade BI-RADS 4a lesions at a cutoff of 2.55 m/s or less (= 19.5 kPa or less) could result in a 24% reduction of false positives (and therefore unnecessary biopsies) while maintaining cancer detection rates consistent with current guidelines [6]. In contrast to these two previous studies, the study by Xu et al is the first prospective multicenter study to use 3D shear-wave elastography. While the reported diagnostic performance of 3D shear-wave elastography is descriptively higher compared to 2D shear-wave elastography, measurement reliability is (currently) slightly lower. Although the widespread adoption of 3D elastography may improve its reliability, it remains unclear whether to recommend 2D or 3D systems. Nonetheless, the study by Xu et al adds to the growing evidence that breast elastography improves conventional breast ultrasound. However, two issues remain:

1. To date, no trial could prospectively confirm the cutoffs to up- or downgrade suspicious breast masses which are solely based on exploratory analyses conducted in retrospect (and which are remarkably different among the three large trials mentioned above). Change of practice is difficult to be based on hypothetical re-classifications.
2. Variations in vendors and interpretation techniques continue to represent a challenge for users and scientific societies. Further standardization is needed in order to recommend breast elastography on a large scale.

Novel research in the field of breast elastography is ongoing: Recently, an improved software has been evaluated that showed a drastic increase in diagnostic performance in a prospective single-center study [7]. Other studies by Pfob et al are evaluating the use of artificial intelligence in breast elastography with promising results [8]. Although breast elastography may not (yet) be ready for prime time, we need high-quality studies in line with previously published ones [4–6] to drive innovation and research within the imaging community and to ultimately improve clinical care.

## Declarations

### Guarantor

The scientific guarantor of this publication is André Pfob.

### Statistics and biometry

No complex statistical methods were necessary for this paper.

### Methodology

• commentary
